# Unexpected skin lesions secondary to metastasis of urothelial carcinoma

**DOI:** 10.1016/j.ijscr.2019.10.007

**Published:** 2019-10-11

**Authors:** Tarek Taktak, Hamza Boussaffa, Yassine Ouanes, Selim Zaghbib, Ahmed Sellami, Zinet Ghorbel, Ines Chelly, Sami Ben Rhouma, Yassine Nouira

**Affiliations:** a Urology Department, La Rabta Teaching Hospital, Faculty of Medicine of Tunis, Tunis El Manar University, Tunis, Tunisia; b Department of Anatomopathology, La Rabta Teaching Hospital, Faculty of Medicine of Tunis, Tunis El Manar University, Tunis, Tunisia

**Keywords:** Skin metastasis, Neoplasms, Urothelial bladder carcinoma, Metastasis

## Abstract

**Introduction:**

Metastatic spread of urothelial bladder carcinoma (UBC) rarely involves the skin which is associated with a poor prognosis. We present a rare case of UBC with cutaneous metastases which is exceptional by its inflammatory clinical form.

**Presentation of case:**

A 62-year-old male was diagnosed with a non-metastatic muscle invasive transitional cell bladder carcinoma invading the anterior wall of the rectum. Cisplatin-based chemotherapy was indicated but refused by the patient. Three months later, he developed cutaneous lesions in the left axilla and the right inguinal fold. These lesions were budding, nodular and inflammatory corresponding to carcinomatous metastasis on skin biopsy which urothelial origin was confirmed by immunohistochemical analysis. The patient died four weeks later after multi-organ failure.

**Discussion:**

Skin metastasis of transitional cell carcinoma of the bladder are uncommon, representing 0.84% of all cutaneous metastases. The inflammatory presentation, as seen in our case, is rarer than the other types and is usually due to a lymphatic extension. The clinical appearance of cutaneous metastases might mimic other common dermatologic disorders; Thus, diagnosis requires histological confirmation by microscopic examination and immunohistochemical study of a skin biopsy. The prognosis after the appearance of cutaneous metastases is generally poor with a median disease-specific survival of less than 12 months Treatment is palliative and is principally based on chemotherapy, analgesics and psychological support.

**Conclusion:**

Cutaneous metastases secondary to urothelial bladder carcinoma are exceptional especially in its inflammatory presentation. Diagnosis is based on immunohistochemical study. Treatment is based on chemotherapy and the prognosis is poor.

## Highlights

• Skin metastases of urothelial bladder carcinoma are rare.
• The inflammatory presentation, as seen in our case, is exceptional.
• Diagnosis requires immunohistochemical study of a skin biopsy.
• The prognosis after the appearance of cutaneous metastasis is poor.

## 1 Introduction

Metastatic spread of urothelial bladder carcinoma rarely involves the skin [1]. However, the presence of skin metastasis indicates at least microscopic dissemination of neoplastic cells to the lung or liver, corresponding to a poor prognosis [2]. We present a rare case of urothelial bladder carcinoma with cutaneous metastases to the left axillary region and the right inguinal fold that is exceptional by its inflammatory clinical form [3]. Our work has been reported in line with the SCARE criteria [4].

## 2 Presentation of case

A 62-year-old male patient has complained of hematuria and pollakiuria for 6 months. Digital rectal examination found a fixed bladder floor. Ultrasound showed a bladder tumor measuring 5 cm. Cystoscopy revealed papillary tumor of the right lateral wall of the bladder. Transurethral resection of tumor was performed and histopathological examination revealed a muscle invasive transitional cell bladder carcinoma of high grade. Computerized tomography showed a large tumor of the bladder right wall invading peri vesical fat and the anterior wall of the rectum. Upper urinary tract was normal and there was no hepatic pulmonary or bone metastasis.

**Fig. 1:**
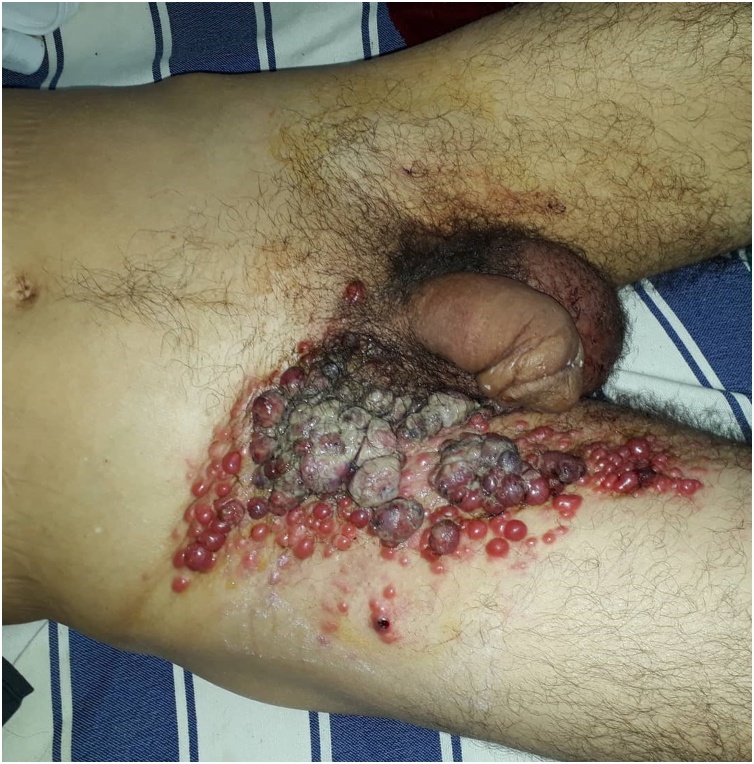
Nodular, budding and inflammatory skin lesions of the right inguinal fold.

**Fig. 2:**
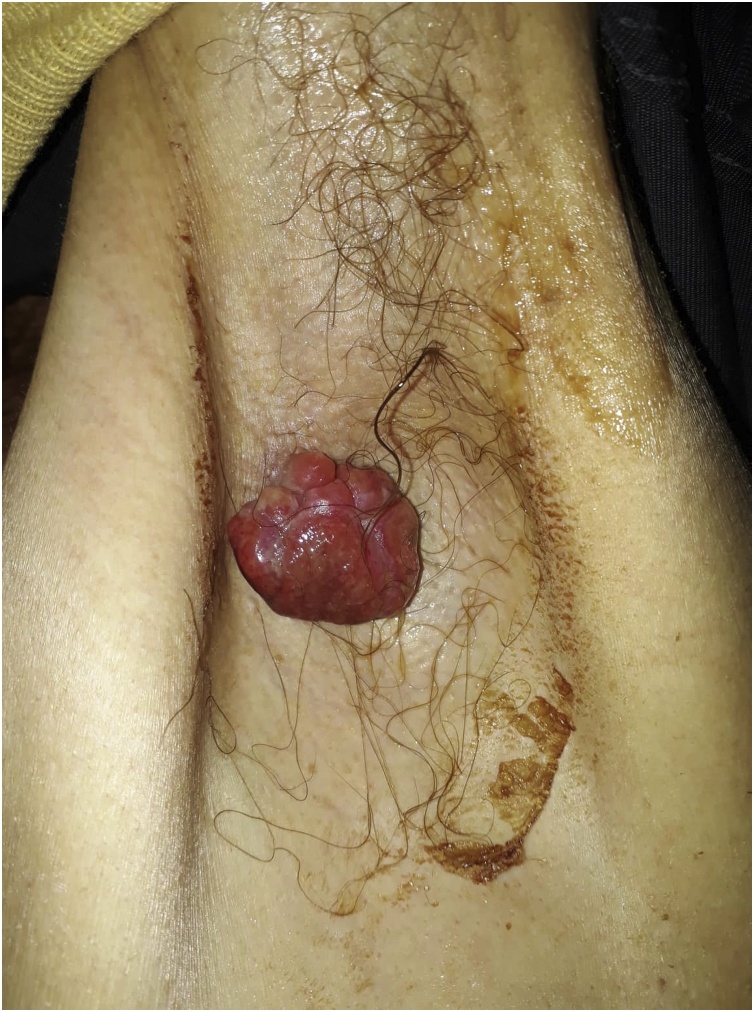
A papillary skin lesion of the left axilla.

Cisplatin-based chemotherapy was indicated but refused by the patient. Three months later, he presented to our department for skin lesions in the right inguinal fold (Fig. 1) and the left axilla (Fig. 2). These lesions were budding, nodular, inflammatory, firm to palpation and some of them were infected. A paracetamol and codeine association was prescribed to the patient along with a local antiseptic.

**Fig. 3:**
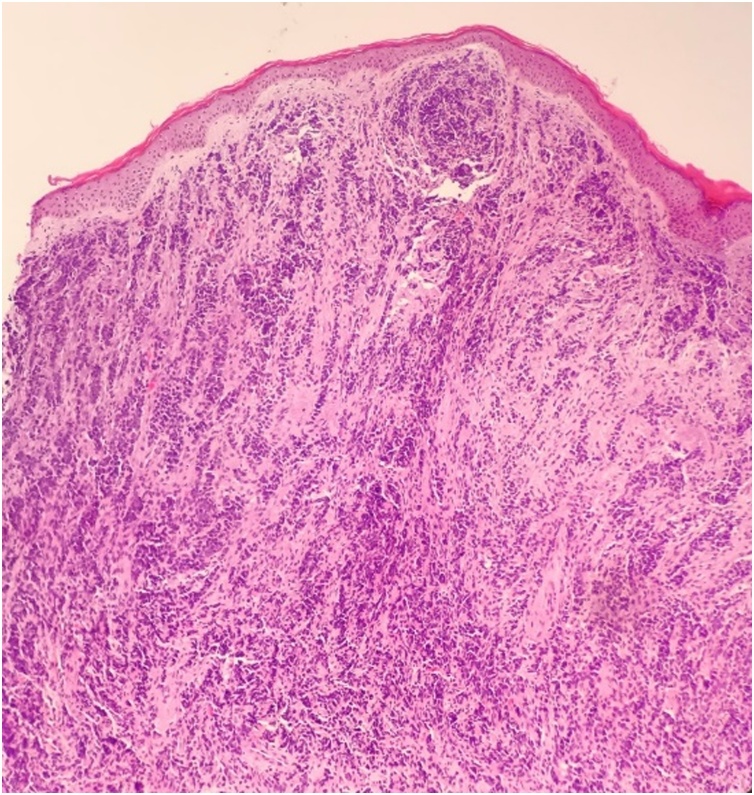
HE X 20: poorly differentiated carcinomatous proliferation with diffuse layers of cells and trabecular growth pattern that occupies the entire dermis.

**Fig. 4:**
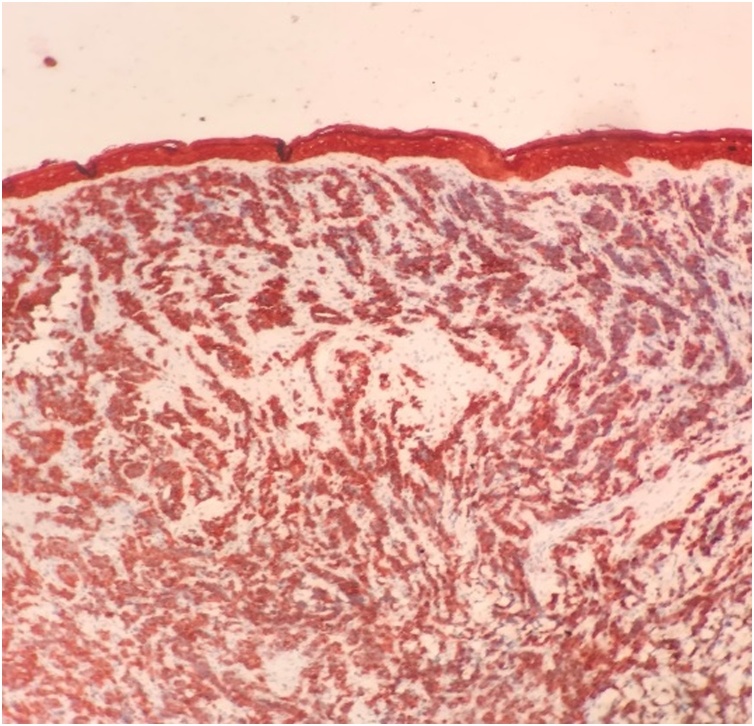
Positive staining of tumor cells with Cytokeratin.

Skin biopsy of the inguinal lesion was performed, showing poorly differentiated carcinomatous proliferation with diffuse layers of cells and trabecular growth pattern that occupies the entire dermis (Fig. 3). The immunohistochemical analysis showed p63 and cytokeratin positive staining, confirming urothelial origin (Fig. 4). The patient refused categorically chemotherapy. His condition rapidly deteriorated, and death occurred four weeks later after multi-organ failure.

## 3 Discussion

Skin metastasis of transitional cell carcinoma of the bladder are uncommon, representing 0.84% of all cutaneous metastases [1]. It may occur as a result of direct tumor invasion, hematogenous or lymphatic spread, or as a result of iatrogenic implantation of tumor cells [5]. Clinical features of skin metastasis are divided into three presentations: nodular, inflammatory and sclerodermoid [3]. The inflammatory presentation, as seen in our case, is rarer than the other types and is usually due to a lymphatic extension causing tumor thrombi in the lymphatic vessels of the skin and dermis invasion [2,3]. In our case, we believe that it was secondary to lymphatic spread through the thoracic lymphatic duct explaining the left axillary location. However, the inguinal lymph nodes are not the usual drainage territory of bladder which may be in favor of hematogenous spread.

The clinical appearance of cutaneous metastases might mimic other common dermatologic disorders; Thus, diagnosis requires histological confirmation by microscopic examination and immunohistochemical study of a skin biopsy which confirms the urothelial origin of these lesions [6].

The prognosis after the appearance of cutaneous metastases is generally poor, as it indicates at least microscopic dissemination of neoplastic cells to the lung or liver, with a median disease-specific survival of less than 12 months but cases of longer-term survival up to 34 months have been reported [2].

Treatment is palliative and is principally based on chemotherapy, analgesics and psychological support. However, some authors conducted local skin radiation in addition to cisplatin-based chemotherapy, with complete regression of the skin lesions [7].

## 4 Conclusion

Cutaneous metastases secondary to urothelial bladder carcinoma are exceptional, especially in the absence of other metastatic sites. Diagnosis is based on immunohistochemical study of a skin biopsy. Treatment is based on chemotherapy and the prognosis is poor.

## Funding

No source of funding.

## Ethical approval

La Rabta Teaching Hospital ethic comitee, Tunis, Tunisia.

## Consent

Written informed consent was obtained from the patient for publication of this case report and accompanying images.

## Author contribution

Taktak T; concept or design, data collection, data analysis or interpretation, writing the paper.

Boussaffa H; concept or design, data collection, data analysis or interpretation, writing the paper.

Zaghbib S; concept or design, data collection, data analysis or interpretation, writing the paper.

Ouanes Y; data collection, data analysis or interpretation.

Chelly I; data collection and data analysis.

Ghorbel Z; histological examination.

Sellami A; data analysis and interpretation.

Ben Rhouma S; data collection, data analysis or interpretation, writing the paper.

Nouira Y; writing the paper.

## Research studies

This is no research study.

## Guarantor

Taktak Tarek.

## Provenance and peer review

Not commissioned, externally peer-reviewed.

No financial and personal relationships with other people or organisations that could inappropriately influence (bias) their work.

## Declaration of Competing Interest

No financial and personal relationships with other people or organisations that could inappropriately influence (bias) their work.
